# Reconstruction of Severe Acetabular Bone Defect with 3D Printed Ti6Al4V Augment: A Finite Element Study

**DOI:** 10.1155/2018/6367203

**Published:** 2018-11-14

**Authors:** Jun Fu, Ming Ni, Jiying Chen, Xiang Li, Wei Chai, Libo Hao, Guoqiang Zhang, Yonggang Zhou

**Affiliations:** Department of Orthopaedics, Chinese People's Liberation Army General Hospital (301 Hospital), Beijing, China

## Abstract

**Purpose:**

The purpose of this study was to establish the finite element analysis (FEA) model of acetabular bone defect reconstructed by 3D printed Ti6Al4V augment and TM augment and further to analyze the stress distribution and clinical safety of augments, screws, and bones.

**Methods:**

The FEA model of acetabular bone defect reconstructed by 3D printed Ti6Al4V augment was established by the CT data of a patient with Paprosky IIIA defect. The von Mises stresses of augments, screws, and bones were analyzed by a single-legged stance loading applied in 3 increments (500 N, 2000 N, and 3000 N).

**Results:**

The peak von Mises stresses under the maximal loading in the 3D printed augments, screws, and cortical bone were less than the yield strength of the corresponding component. However, the peak stress in the bone was greater than the yield strength of cancellous bone under walking or jogging loading. And under the same loading, the peak compressive and shear stresses in bone contact with TM augment were larger than these with 3D printed augment.

**Conclusions:**

The FEA results show that all the components will be intact under single-legged standing. However, partial cancellous bone contacted with 3D printed augment and screws will lose efficacy under walking or jogging load. So we recommend that patients can stand under full bearing, but can not walk or jog immediately after surgery.

## 1. Introduction

Total hip arthroplasty (THA) has been one of the most successful surgeries in the 20^th^ century, which is used to release pain, correct deformity, and improve function of the hip joint [1]. The management of severe acetabular bone defects in primary or revision THA is challenging and the ideal reconstruction of them is one of the critical success factors in THA. The basic principles of acetabular defect reconstruction include restoring hip center of rotation (HCOR) and acetabular ring integrity, preserving acetabular bone stock and establishing normal biomechanics of the hip, which could obtain immediate and long-term stability of acetabular components [2–4]. Given the biocompatibility and biomechanical properties, titanium alloys (Ti6Al4V) particularly with a porous coating are widely used to fabricate orthopaedic prosthesis and instruments [5–8].

Over the past few decades, finite element analysis (FEA) has been utilized with enormous success in orthopaedics field to analyze problems such as bone remodeling, fracture healing, implant design, and the interactions at the bone-implant interface. Levine et al. [9] applied FEA to analyze the biomechanical changes of acetabular defect repaired by a Trabecular Metal (TM) augment. And the results showed the use of a TM augment provided structural stabilization and helped to restore HCOR. However, the acetabular bone defects in their study were created to match the shape and size of the TM augment. Real defects were irregular shape and would impact the stress locally.

The purpose of this study was to establish an integrated FEA model of acetabular bone defect reconstructed by the 3D printed Ti6Al4V augment and TM augment, which included acetabular and femoral components, ceramic insert and femoral head, augment, and screws. After that the stress distribution and clinical safety of augments, screws, bone, and prosthesis were analyzed under different loads.

## 2. Methods

The FEA model was based upon the geometry of a human pelvis, obtained from the CT scan of a female patient (bodyweight, BW: 50 kg) with Paprosky IIIA acetabular bone defect, who signed an informed consent for this study. The three-dimensional reconstruction model of the pelvis was generated using Mimics Research 20.0 (Materialise, Belgium). And then the 3D printed Ti6Al4V augment was designed using the computer aided design (CAD) software-Creo 2.0 (PTC, America). The 3D printed Ti6Al4V augment had a 1 mm layer of porous coating of the same material and the rest of the augment was solid. The STL data of designed augment and pelvis was used to conduct the reverse engineering reconstruction using Geomagic 2012 (Geomagic, America), which included segmentation, smoothing, polishing, denoising, and other image processing of the three-dimensional model. Then the solid model was generated into IGES three-dimensional image.

**Figure 1:**
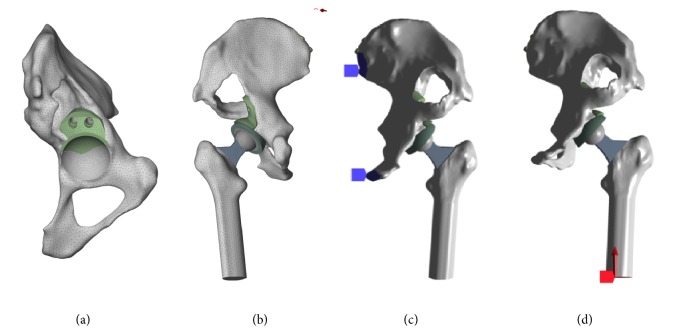
(a) The 3D printed augment was put on the acetabular defect surface fixed with two screws. (b) The general layout with all components and mesh generation was made after setting up all the material properties and interfaces. (c) Fixed constraint boundary conditions were assumed at the sacroiliac joint and pubic symphysis. (d) Simulated vertical reaction load was applied from the bottom of femur.

According to actual size of the patient's acetabulum and femur, acetabular and femoral components were assembled. Acetabular cup was 46 mm porous tantalum coating cup (Zimmer, America) with ceramic insert (Zimmer, America). Femoral prosthesis was size 9 LCU (Link, Germany) with 28-3.5 mm ceramic femoral head (Link, Germany). Then the 3D printed augment was placed on the acetabular defect surface fixed with two screws (the diameter of screw was 6.5 mm and the length was determined by the residual bone stock). After that, the gap between cup and augment was filled with bone cement (Figure 1(a)).

Then the solid model was imported into Ansys Workbench 16.2 (Ansys, America) and Boolean operation was performed. Mesh generation was made after setting up all the material properties and interfaces. The mesh size was set as 1 mm using an automatic mesh technique, as validated in a previous study [10]. Element type was chosen as Solid 187, and a total of 290065 elements and 495201 nodes were generated. Material properties used [11–14] in the model are presented in Table 1. Tied contact was assumed at the interfaces between the components except between ceramic insert and femoral head (friction coefficient was 0.06 [15]).

A resultant equivalent load (single-legged stance loading) was applied, taking account of muscles damaged around the hip joint in most of the revision THA patients. The peak force measurements for unilateral hip joint were reported in the majority of literature showing 100%BW (500 N) for single-legged standing, 400%BW (2000 N) for walking, and 600%BW (3000 N) for jogging [16, 17]. Fixed constraint boundary conditions were assumed at the sacroiliac joint and pubic symphysis. The femur was constrained in all directions at the mid-diaphysis and the simulated vertical reaction load was applied from the bottom of femur at 500 N, 2000 N, and 3000 N. Figure 1 shows the general layout used for these FEA models and the details of components, load, interfaces, and constraint boundary. The main analysis was the local stress of augments, screws, acetabular bone, and the other components.

## 3. Results

### 3.1. Stress Distribution of the 3D Printed Ti6Al4V Augment

**Figure 2:**
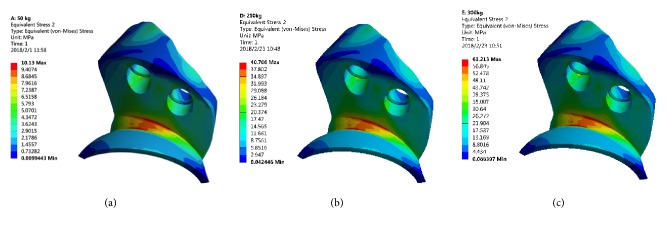
The stress distribution of 3D printed Ti6Al4V augment. (a) 500 N; (b) 2000 N; (c) 3000 N.

The peak von Mises stress in the 3D printed Ti6Al4V augment was located in the dome connection of the augment and the arc cup, which was 10.13 MPa (500 N), 40.71 MPa (2000 N), and 61.21 MPa (3000 N), respectively (Figure 2).

### 3.2. Stress Distribution of the Screw for Fixing 3D Printed Ti6Al4V Augment

**Figure 3:**
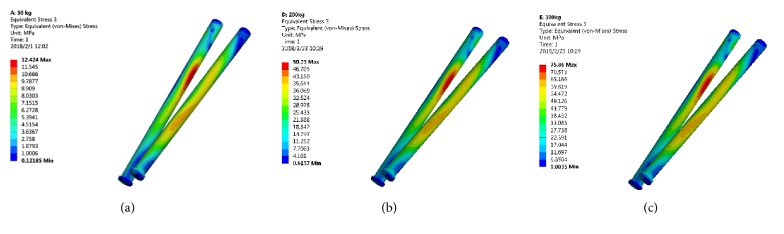
The stress distribution of screw for fixing 3D printed Ti6Al4V augment. (a) 500 N; (b) 2000 N; (c) 3000 N.

The peak von Mises stress in the screw for fixing 3D printed Ti6Al4V augment was located in the lower 1/3 of the screw, which was 12.42 MPa (500 N), 50.25 MPa (2000 N), and 75.86 MPa (3000 N), respectively (Figure 3).

### 3.3. Stress Distribution of the Bone in Contact with 3D Printed Ti6Al4V Augment

**Figure 4:**
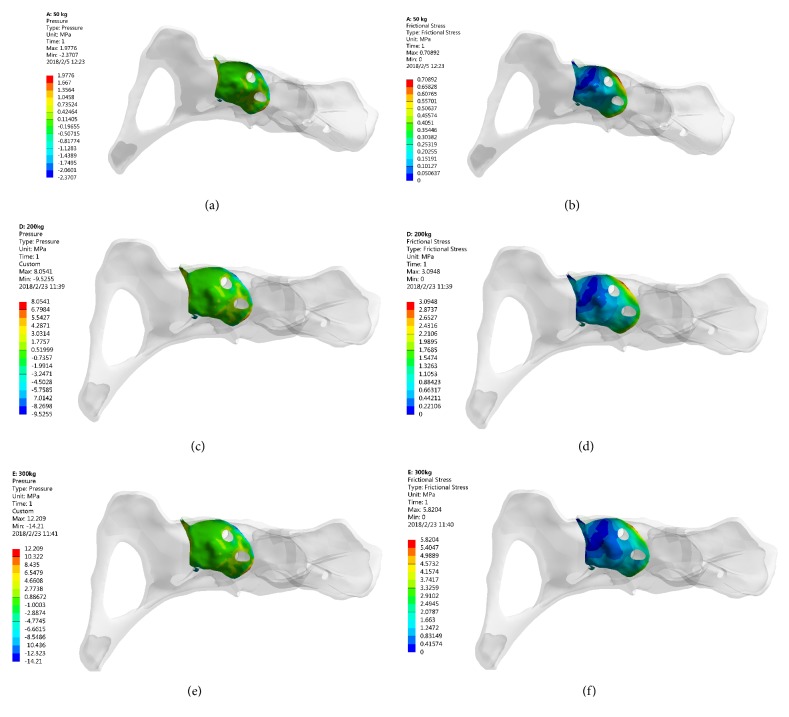
The stress distribution of bone in contact with 3D printed Ti6Al4V augment. (a-b) 500 N; (c-d) 2000 N; (e-f) 3000 N.

The peak stresses in the bone interface in contact with the 3D printed Ti6Al4V augment were divided into compressive stress and shear stress, located at the edge of contacted bone. And the peak stress values of compressive and shear were 2.37 MPa and 0.71 MPa (500 N), 9.53 MPa and 3.09 MPa (2000 N), and 14.21 MPa and 5.82 (3000 N), respectively (Figure 4).

### 3.4. Stress Distribution of the Bone in Contact with Screw

**Figure 5:**
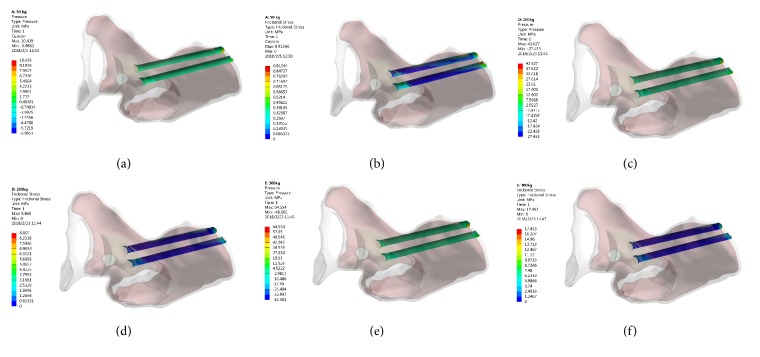
The stress distribution of bone in contact with screw. (a-b) 500 N; (c-d) 2000 N; (e-f) 3000 N.

The peak stresses in the bone interface in contact with screw were also divided into compressive stress and shear stress, located at the end of screw with contacted cortical bone. And the peak stress values were 10.44 MPa and 0.91 MPa (500 N), 42.63 MPa and 8.87 MPa (2000 N), and 64.55 MPa and 17.45 (3000 N), respectively (Figure 5).

### 3.5. Stress Distribution of the Bone in Contact with TM Augment

**Figure 6:**
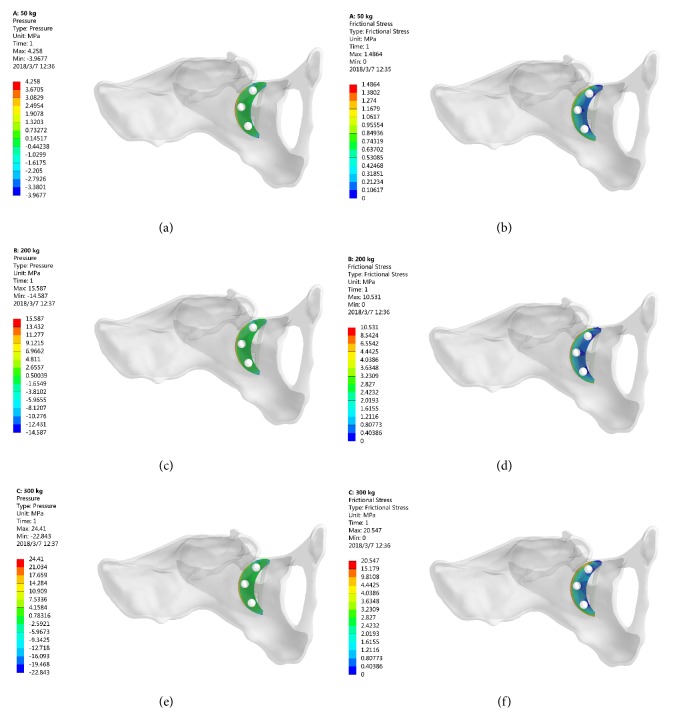
The stress distribution of bone in contact with TM augment. (a-b) 500 N; (c-d) 2000 N; (e-f) 3000 N.

**Figure 7:**
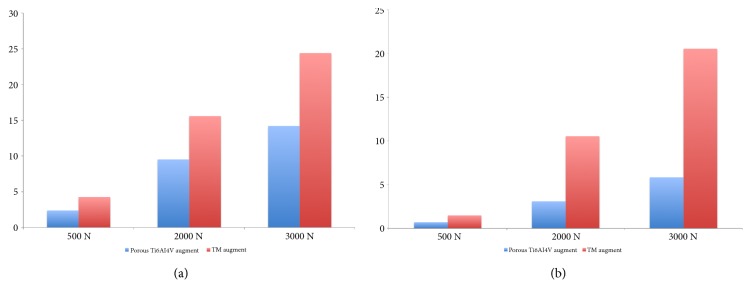
The comparison of bone stress in 3D printed Ti6Al4V augment and TM augment. (a) Compressive stress; (b) shear stress.

The peak stresses in the bone interface in contact with TM augment divided into compressive stress and shear stress were located at the edge of contacted bone. And the peak stress values were 4.26 MPa and 1.49 MPa (500 N), 15.59 MPa and 10.53 MPa (2000 N), and 24.41 MPa and 20.55 MPa (3000 N), respectively (Figure 6). And the peak compressive and shear stresses in bone contact with TM augment were larger than these with 3D printed Ti6Al4V augment (Figure 7).

### 3.6. Stress Distribution of the Other Components

The peak von Mises stresses of the other components were less than the yield strength of the materials, including the acetabular cup and insert, the ceramic femoral head, and the femoral prosthesis.

## 4. Discussion

This current study of three-dimensional FEA model of a patient with Paprosky IIIA acetabular bone defect was constructed from CT scan data and used to examine the stress changes of acetabular bone defect reconstructed with 3D printed Ti6Al4V augment and TM augment when the system is subjected to static loads.

The main bone defect of Paprosky IIIA was the superior rim of acetabulum with superolateral migration (>3 cm) of the hip center and moderate teardrop and ischial osteolysis, which seriously affected the hip joint function and patient's life quality. Therefore, a surgical reconstruction in this case should not only match physiologic stress and transfer mechanical load but also restore the HCOR and hip joint function. Figure 1 showed that the 3D printed Ti6Al4V augment and TM augment provided a buttress support with stable fixation, which maintained the acetabular cup in a good position that restored the HCOR and limb-length discrepancy (LLD). However, in order to match the shape of TM augment, the defect bone surface should be reamed and the remaining bone stock was further compromised.

All materials in our study were modeled as continuum solids with averaged isotropic material properties. Furthermore, the 3D printed Ti6Al4V augment had only a 1 mm porous coating and therefore the elastic modulus of titanium alloy was adopted. The femoral prosthesis (LCU) had a hydroxylapatite coating and the elastic modulus of it was also defined as titanium alloy and the acetabular cup and TM augment were porous tantalum and acceptable for bulky solid components as tantalum.

Yoshida H et al. [18] conducted a three-dimensional dynamic hip FEA model and analyzed the stress distribution during activities of daily living. Their research results showed the peak contact stresses corresponding to the beginning of the mid-stance of gait and at this point in the gait cycle, the entire body weight is supported by only one leg. The average patient loaded the hip joint with 81%BW when standing on one leg, 238-390%BW for normal walking, and 500-600%BW for jogging [16, 17, 19]. The muscles around the hip joint were subject to different degrees of damage in major revision THA patients. In order to identically analyze the stress distribution, the FEA model was simplified to take no account of muscles and applied under single-legged stance loading. Furthermore, the load values in our study were set as 500 N (100%BW for single-legged standing), 2000 N (400%BW for normal waling), and 3000 N (600%BW for jogging).

A few previous studies have researched the FEA models of acetabular or periacetabular bone defects. Li et al. [20] investigated the effects of periacetabular lesion size, cortex involvement, and cement modulus on periacetabular bone stresses under single-legged stance loading. Their results showed that cortical bone stresses were more profoundly affected in the presence of transcortical defects and that cement filling with a modulus of 2.2 GPa was shown to restore cortical bone stresses to near intact values. Kaku et al. [21] evaluated the maximum stress generated on the Kerboull-type (KT) plate and screws using 12-pattern FEA models and larger bone defects increased the stress on the KT plate and screws. Amirouche F et al. [22] conducted a FEA model to evaluate cup insertion and fixation in the context of segmental acetabular defects and they demonstrated defect in the columns creating cup instability and increased stress at the defect location.

This study focused on whether the stresses distribution of 3D printed Ti6Al4V and TM augments, screws for fixing augments, and acetabular bone were greater than the yield strength of the corresponding component. The 3D printed Ti6Al4V augment and screws were titanium alloy and the reported yield strength of titanium alloy was 889-921 MPa [23]. Under maximal loading (3000 N) in our study, the peak von Mises stress of 3D printed Ti6Al4V augment and screws was 61.21 MPa (Figure 2(c)) and 75.86 MPa (Figure 3(c)), respectively, which was far less than the yield strength of titanium alloy. Therefore, the 3D printed Ti6Al4V augment and screws would not lose efficacy even under maximal loading immediately after surgery.

Previous studied reported the mean yield strength of cancellous bone and cortical bone was 3.3 MPa and 93.4 MPa, respectively [24, 25]. Under 500 N loading (single-legged standing), the peak stresses of cancellous bone interface in contact with 3D printed Ti6Al4V augment and screws were 1.05 MPa and 0.782 MPa, while the peak stresses of cortical bone interface in contact with 3D printed Ti6Al4V augment and screws were 2.37 MPa and 10.44 MPa. Although these stresses are not directly related to the yield of bone, they were less than the yield strength of cancellous and cortical bones. That is to say, the periacetabular bone strength was enough to support this patient's single-legged standing.

Under 2000 N loading (normal walking), the peak stress of cancellous bone interface was 4.29 MPa (in contact with 3D printed Ti6Al4V augment) and the peak stress of cortical bone was 42.63 MPa (in contact with screws). Under 3000 N loading (jogging), the peak stresses of cancellous and cortical bone interface were 6.55 MPa (in contact with 3D printed Ti6Al4V augment) and 64.55 MPa (in contact with screws). The peak stress value was greater than the yield strength of cancellous bone but less than the yield strength of cortical bone. As a result, the acetabular cancellous bone interface attached with 3D printed Ti6Al4V augment and screw might lose some degree of efficacy under walking or jogging load immediately after surgery.

The acetabular bone defect model in Levine's study [9] was computerized according to the shape of TM augment, which is not the real shape of bone defect in clinical cases. The acetabular bone defect model in our study was based on a real patient with Paprosky IIIA defect. And to match the shape of TM augment, the defect bone surface in our study had to be reamed. In order to analyze the advantages of 3D printed Ti6Al4V augment, TM augment was defined as the controlled group to reconstruct the same acetabular bone defect and to evaluate the stress distribution of components. Under the same loading, the peak compressive and shear stresses in bone contact with TM augment were larger than these with 3D printed Ti6Al4V augment.

## 5. Conclusion

The current FEA-based study was conducted to build a foundation for a biomechanical rationale that provided a treatment option of reconstruction of acetabular bone defect. The FEA results showed that all the components were intact under single-legged standing. However, partial cancellous bone interface contacting 3D printed augment and screws might lose some degree of efficacy under walking or jogging load. So we recommend that patients can stand under full bearing, but can not walk or jog immediately after surgery.

## Figures and Tables

**Table 1:** Mechanical properties of materials used in FEA mode.

**Components**	**Materials**	**Elastic Modulus (MPa)**	**Poisson's ratio**
Cortical bone	Cortical bone	17300	0.265

Cancellous bone	Cancellous bone	400	0.2

Screws	Titanium alloy	110600	0.326
3D printed augment			
Femoral prosthesis			

Acetabular cup	Tantalum	8963	0.31
TM augment			

Ceramic insert	Ceramics	350000	0.22
Ceramic femoral head			

## Data Availability

The data used to support the findings of this study are included within the article.
